# Risk Factors for Infection after 46,113 Intramedullary Nail Operations in Low- and Middle-income Countries

**DOI:** 10.1007/s00268-012-1817-4

**Published:** 2012-10-02

**Authors:** Sven Young, Stein Atle Lie, Geir Hallan, Lewis G. Zirkle, Lars B. Engesæter, Leif I. Havelin

**Affiliations:** 1 Department of Orthopaedic Surgery, Haukeland University Hospital, Bergen, Norway; 2 Department of Surgical Sciences, University of Bergen, Bergen, Norway; 3 Department of Surgery, Kamuzu Central Hospital, Lilongwe, Malawi; 4 Norwegian Arthroplasty Register, Bergen, Norway; 5 SIGN Fracture Care International, Richland, WA USA

## Abstract

**Background:**

The fields of surgery and trauma care have largely been neglected in the global health discussion. As a result the idea that surgery is not safe or cost effective in resource-limited settings has gone unchallenged. The SIGN Online Surgical Database (SOSD) is now one of the largest databases on trauma surgery in low- and middle-income countries (LMIC). We wished to examine infection rates and risk factors for infection after IM nail operations in LMIC using this data.

**Methods:**

The SOSD contained 46,722 IM nail surgeries in 58 different LMIC; 46,113 IM nail operations were included for analysis.

**Results:**

The overall follow-up rate was 23.1 %. The overall infection rate was 1.0 %, 0.7 % for humerus, 0.8 % for femur, and 1.5 % for tibia fractures. If only nails with registered follow-up (*n* = 10,684) were included in analyses, infection rates were 2.9 % for humerus, 3.2 % for femur, and 6.9 % for tibia fractures. Prophylactic antibiotics reduced the risk of infection by 29 %. Operations for non-union had a doubled risk of infection. Risk of infection was reduced with increasing income level of the country.

**Conclusions:**

The overall infection rates were low, and well within acceptable levels, suggesting that it is safe to do IM nailing in low-income countries. The fact that operations for non-union have twice the risk of infection compared to primary fracture surgery further supports the use of IM nailing as the primary treatment for femur fractures in LMIC.

## Introduction

Approximately 5.8 million people die annually as the result of injuries, more people than die of HIV/AIDS, tuberculosis, and malaria combined [1, 2]. Over 90 % of these fatal injuries occur in low- and middle-income countries (LMIC). For every death from injury, 3–10 more people survive injury with a permanent disability [3, 4]. In young people between the ages of 10 and 24 years as many as 97 % of deaths occur in LMIC, over 40 % of deaths are related to injuries, and road traffic injuries are the most common cause [5]. The global burden of injuries is growing rapidly, and almost entirely in LMIC. By 2030 the World Health Organization (WHO) expects traffic accidents to have risen from the ninth to the fifth leading cause of all deaths globally [6]. Despite these compelling facts, surgery is not mentioned at all in the Millennium Development Goals (MDGs: http://www.un.org/millenniumgoals/) [7, 8]. At present, however, we are seeing increasing awareness of surgery as an integral part of the global public health effort to reach the MDGs [8–12]. Injuries disproportionately affect the younger segment of the population in LMIC and have a serious impact on the whole families of the injured. In LMIC with no functioning social security systems, the injury of a young mother or father, often the breadwinner of the family, can be devastating to their economic situation and push them further into poverty [13].

In high-income countries intra-medullary (IM) nailing of femoral shaft fractures is an established gold standard. However, the cost of IM nails and the fear of postoperative infection has prohibited their use in most LMIC, where traction most often still is the only treatment offered for femoral fractures [14, 15]. In orthopedic surgery, skills and training are useless without the equipment to do the job. This is recognized by SIGN Fracture Care International (SIGN) which has developed a US Food and Drug Administration (FDA) approved IM nail specifically designed for use in resource- poor settings without the use of an image intensifier [16]. SIGN has provided over 80,000 IM nails, and training in their use, to over 200 hospitals in LMIC free of charge since 1999 (numbers; personal communication from SIGN, February 2012) [17]. Although there is growing evidence that orthopedic trauma surgery is necessary, safe, and cost-effective also in LMIC [18–21], more research is needed to confirm these findings and to bring this knowledge into the mainstream global health discussion. As a part of the resupply service for the hospitals supported by SIGN, the SIGN online surgical database (SOSD) was started in 2003. There are now over 46,000 registered IM nail operations in this database, making it possibly the largest available database on orthopedic trauma care in LMIC. Despite a fairly limited follow-up rate (18.1 % in 2010), validation of the data in the SOSD has suggested that it is reliable and can be used for further research [21]. The aim of the present study was to use the data in the SOSD to investigate whether the follow-up and infection rates are changing, and to identify risk factors for infection after IM nail operations in LMIC.

## Methods

SIGN provided us with an anonymous export of all surgeries registered in the SOSD from the start of the registry in 2003 up to November 29, 2011. Ethical approval for this study was given by the Norwegian regional research ethics committee (20.09.10, 2010/2040). The SOSD at the time of export contained data on 46,722 IM nail operations. 562 operations were for hip fractures or did not have a registered surgical approach and were excluded. As only 47 operations were done in high-income countries (USA 38, Australia 9), and only one of these cases had a registered follow-up, these cases were also excluded. This left 46,113 IM nail operations of the humerus, femur, or tibia to be included for analysis. An overview of the included cases and risk factors is presented in Table 1.

**Table 1:** Overview of included cases in the SIGN online surgical database

	Number of operations (%)	Number with follow-up (rate in %)	Number of open fractures (rate in %)	Number of infections (rate in %)
Included operations in SOSD	46,113 (100)	10,684 (23.1)	7,831 (17.0)	479 (1.0)
Age				
<30 years	20,896 (45.3)	5,029 (24.1)	3,822 (18.3)	216 (1.0)
≥30 years	25,217 (54.7)	5,655 (22.4)	4,009 (15.9)	263 (1.0)
Gender				
Female	8,664 (18.8)	2,213 (25.5)	1,080 (12.5)	71 (0.8)
Male	37,449 (81.2)	8,471 (22.6)	6,751 (18.0)	408 (1.1)
Approach				
Antegrade humerus	1,381 (3.0)	310 (22.4)	110 (8.0)	9 (0.7)
Antegrade femur	17,450 (37.8)	4,355 (25.0)	1,431 (8.2)	130 (0.7)
Retrograde femur	9,900 (21.5)	2,292 (23.2)	1,230 (12.4)	84 (0.8)
Tibia	17,382 (37.7)	3,727 (21.4)	5,060 (29.1)	256 (1.5)
Prophylactic antibiotics				
No	6,538 (14.2)	8,666 (21.9)	7,478 (18.9)	78 (1.2)
Yes	39,575 (85.8)	2,018 (30.9)	353 (5.4)	401 (1.0)
Fracture reduction				
Closed	12,216 (26.5)	8,314 (19.4)	5,814 (17.1)	102 (0.8)
Open	33,897 (73.5)	2,370 (24.5)	2,017 (16.5)	377 (1.1)
Reaming method				
None	3,996 (8.7)	472 (11.8)	1,169 (29.2)	31 (0.8)
Hand	41,593 (90.2)	10,033 (24.1)	6,592 (15.8)	440 (1.1)
Power	524 (1.1)	179 (34.2)	70 (13.3)	8 (1.5)
Operation for non-union				
No	41,441 (89.9)	1,350 (28.9)	470 (10.0)	379 (0.9)
Yes	4,672 (10.1)	9,334 (22.5)	7,361 (17.8)	100 (2.1)
Gustilo–Anderson grade				
Closed	38,297 (83.1)	8,881 (23.2)	–	293 (0.8)
Open grade 1	2,777 (6.0)	595 (21.4)	2,777 (6.0)	40 (1.4)
Open grade 2	2,936 (6.4)	681 (23.2)	2,936 (6.4)	69 (2.4)
Open grade 3a	1,562 (3.4)	383 (24.5)	1,562 (3.4)	48 (3.1)
Open grads 3b	467 (1.0)	125 (26.8)	467 (1.0)	24 (5.1)
Open grade 3c	74 (0.2)	19 (25.7)	74 (0.2)	5 (6.8)
Country income level ^a^				
Low-income countries	25,751 (55.8)	7,197 (27.9)	4,192 (16.3)	309 (1.2)
Lower middle- income countries	17,083 (37.0)	3,168 (18.5)	3,231 (18.9)	153 (0.9)
Higher middle-income countries	3,279 (7.1)	319 (9.7)	393 (12.0)	17 (0.5)

^a^Country income level according to World Bank 2009

Infection was registered in the SOSD at the time of follow-up. Possible risk factors for infection after orthopedic trauma surgery, including age, gender, surgical approach, use of antibiotics, and operating techniques were included as variables in the analyses. Open fractures in the SOSD were classified according to Gustilo and Anderson [22]. Surgeons classified infections as superficial or deep in the SOSD; however, this distinction did not follow a strict classification. We therefore grouped all registered infections together on the assumption that registered infections are likely to be clinically significant. The duration of the operative procedure was not registered in the SOSD, but surgeons did subjectively classify a fracture as a non-union or not at the time of surgery. Non-union may be a risk factor in itself, or it might be an indirect measure of increased operating time, and it was therefore analyzed as a separate risk factor. The SIGN IM nail system uses an external target arm and “slot finder” instruments to place the distal locking screws in the nail. This technique can be challenging at times and can prolong operating time. The number of distal locking screws (0, 1, or 2) was therefore also included as another possible indirect measure of operating time.

## Statistics

The χ^2^ test was used to compare rates in two different groups, and Student’s *t* test was used to compare means in two groups. Logistic regression was used to compare rates in more than two groups and to calculate both crude and adjusted risk, odds ratio (OR), of infection. All *p* values were two-tailed, and the level of statistical significance was set to 5 % (*p* < 0.05). Analyses were performed with IBM SPSS Statistics version 19.0 (SPSS Inc., Chicago, IL).

## Results

A total of 46,133 IM nail operations were included, 1,381 operations of the humerus, 27,350 of the femur, and 17,382 of the tibia. Only 18.8 % of operations were in women. The mean age of the patients was 34.7 (SD 15.2) years, 40.6 (SD 18.6) years for women and 33.3 (SD 14.0) years for men (*p* < 0.001).

The total follow-up rate, defined as the percentage of IM nail operations with at least one registered follow-up visit, for all nails registered in the SOSD in November 2011 was 23.1 % (95 % CI: 22.7–23.5), this is an increase from one year before, when the follow-up rate in the SOSD was 18.1 %. The mean time to follow-up was 215 (SD 293) days, median 100 (range: 1–3,309) days. The over all infection rate was 1.0 % (95 % CI: 0.9–1.1); 0.7% (95 % CI: 0.6–0.8) for the humerus, 0.8 % (95 % CI: 0.7–0.9) for the femur, and 1.5 % (95 % CI: 1.4–1.6) for the tibia. Crude and adjusted risks of infection for different risk factors are presented in Table 2. If only nails with registered follow-up (*n* = 10 684) were included, infection rates were 2.9 % for fractures of the humerus (95 % CI: 2.6–3.2), 3.2 % (95 % CI: 2.9–3.5) for those of the femur, and 6.9 % (95 % CI: 6.4, 7.4) for those of the tibia.

**Table 2:** Crude and adjusted risk of infection

	No. operations (%)	No. infections (rate in %)	Crude odds ratio (95 % CI)	*p* value	Adjusted odds ratio (95 % CI)	*p* value
All included operations in SOSD	46,113 (100)	479 (1.0)				
Age (years)						
<30	20,896 (45.3)	216 (1.0)	1		1	
≥30	25,217 (54.7)	263 (1.0)	1.01 (0.84–1.21)	0.92	1.01 (0.84–1.21)	0.96
Gender						
Female	8,664 (18.8)	71 (0.8)	1		1	
Male	37,449 (81.2)	408 (1.1)	1.33 (1.04–1.72)	0.026	1.29 (1.00–1.66)	0.053
Approach						
Antegrade humerus	1,381 (3.0)	9 (0.7)	0.87 (0.44–1.72)	0.70	0.88 (0.45–1.75)	0.72
Antegrade femur	17,450 (37.8)	130 (0.7)	1	<0.001^a^	1	<0.001^a^
Retrograde femur	9900 (21.5)	84 (0.8)	1.14 (0.87–1.50)	0.35	1.13 (0.86–1.50)	0.38
Tibia	17,382 (37.7)	256 (1.5)	1.99 (1.61–2.46)	<0.001	1.71 (1.36–2.15)	<0.001
Prophylactic antibiotics						
No	6,538 (14.2)	78 (1.2)	1		1	
Yes	39,575 (85.8)	401 (1.0)	0.85 (0.66–1.08)	0.19	0.71 (0.55–0.91)	0.008
Fracture reduction						
Closed	12,216 (26.5)	102 (0.8)	1		1	
Open	33,897 (73.5)	377 (1.1)	1.34 (1.07–1.66)	0.010	1.23 (0.97–1.55)	0.083
Reaming method						
None	3,996 (8.7)	31 (0.8)	1	0.14^a^	1	0.14^a^
Hand	41,593 (90.2)	440 (1.1)	1.37 (0.95–1.97)	0.093	1.41 (0.96–2.06)	0.076
Power	524 (1.1)	8 (1.5)	1.98 (0.91–4.34)	0.086	1.92 (0.86–4.25)	0.11
Operation for non-union						
No	41,441 (89.9)	379 (0.9)	1		1	
Yes	4,672 (10.1)	100 (2.1)	2.37 (1.90–2.96)	<0.001	2.31 (1.83–2.91)	<0.001
Gustilo–Anderson grade						
Closed	38,297 (83.1)	293 (0.8)	1	<0.001^a^	1	<0.001^a^
Open grade 1	2,777 (6.0)	40 (1.4)	1.90 (1.36–2.64)	<0.001	1.86 (1.32–2.62)	<0.001
Open grade 2	2,936 (6.4)	69 (2.4)	3.12 (2.40–4.07)	<0.001	2.98 (2.25–3.94)	<0.001
Open grade 3a	1,562 (3.4)	48 (3.1)	4.11 (3.02–5.60)	<0.001	4.00 (2.90–5.50)	<0.001
Open grads 3b	467 (1.0)	24 (5.1)	7.03 (4.59–10.77)	<0.001	6.08 (3.92–9.43)	<0.001
Open grade 3c	74 (0.2)	5 (6.8)	9.40 (3.77–23.47)	<0.001	7.61 (3.01–19.25)	<0.001
Country income level^b^						
Low-income countries	25 751 (55.8)	309 (1.2)	1	<0.001^a^	1	<0.001^a^
Lower middle-income countries	17 083 (37.0)	153 (0.9)	0.74 (0.61–0.90)	0.003	0.71 (0.58–0.86)	0.001
Higher middle-income countries	3,279 (7.1)	17 (0.5)	0.43 (0.26–0.70)	0.001	0.49 (0.30–0.81)	0.005

Crude odds ratio only compares the risk of infection for the particular risk factor in question. The adjusted odds ratio is adjusted for all the other factors in the table

^a^Overall test

^b^Country income level according to World Bank 2009

The crude risk of infection for men was 33 % higher than for women (OR 1.33, 95 % CI 1.04–1.72; *p* = 0.026), but this apparent increased risk marginally lost statistically significance when adjusted for the other risk factors in Table 2 (OR 1.29, 95 % CI 1.00–1.66; *p* = 0.053).

There were 17.0 % open fractures in this study. An open fracture of any grade gave a 3.16 times increased adjusted risk of infection (OR 3.16, 95 % CI 2.62–3.80; *p* < 0.001). The increased risk of infection rose from 1.86 times for a Gustilo type 1 fracture to 7.61 times increased risk for Gustilo type 3c fracture (Table 2). Fractures defined by the surgeon preoperatively as a non-union had an adjusted risk of infection 2.31 times higher (OR 2.31, 95 % CI 1.83–2.91; *p* < 0.001) than fractures that were not classified as a non-union. There was no apparent effect of the number of distal locking screws on the rate of infection (OR 0.95–1.25; *p* = 0.80–0.30), and this variable did not affect the adjusted risks of the other risk factors. It was therefore not included in Table 2. The method of reaming did not significantly affect the risk of infection (Table 2).

The use of prophylactic antibiotics at the time of surgery reduced the adjusted risk of infection by 29 % (OR 0.71, 95 % CI 0.55–0.91; *p* = 0.008). The apparent increase in the crude risk of infection after open reduction (OR 1.34, 95% CI 1.07–1.66; *p* = 0.010) was not statistically significant after adjusting for other risk factors (OR 1.23, 95% CI 0.97–1.55; *p* = 0.083).

Age and gender did not significantly affect the risk of infection on more thorough sub-analysis. The same was the case when analyses were done after exclusion of countries with less than 5 % follow-up, except that the difference in infection risk according to a country’s income level was then no longer present (*p* = 0.68). Sub-analysis was also done after exclusion of all patients without follow-up. This left 10,684 surgeries for analysis. Also here results were mostly unchanged. However, once again the difference in risk of infection according to the income level of the country where the surgery was performed (*p* = 0.30) was no longer statistically significant. In addition the effect of prophylactic antibiotics (*p* = 0.99) was no longer seen on exclusion of patients without follow-up.

## Discussion

The main findings in the present study were that infection rates in the SOSD were low, and that the risk of infection is doubled for the delayed surgery of non-unions. When the results of this study were compared to results from the SOSD one year before [21] an increase in the follow-up rate from 18.1 % (95 % CI: 17.7– 18.5) in 2010 to 23.1 % (95 % CI: 22.7–23.5) in 2011 was observed. Despite this 27.6 % increase in the follow-up rate, the infection rates in the SOSD have not risen notably. The findings that the changes in infection rates are small despite a fairly large increase in follow-up might support the observation many surgeons in low-income countries have made; that a large proportion of the patients who have specific complaints do return for follow-up, whereas patients with no complaints do not return, due, among other things, to the high cost of transport [23]. In the previous article mentioned above, we looked at the effect of the low follow-up rate in the SOSD on the infection rates [21]. In that article the statistical model suggested that the data in the SOSD might support this, as countries registering more than 5 % follow-up had very little difference in infection rates, and no increase in infection rates was found with increasing follow-up rates over 5 %.

The infection rates in the present study are comparable to published infection rates in high-income countries [24–26], even in the higher end of the range [27]. However, there is a widespread belief among surgeons that the risk of postoperative infection is very high in LMIC. This probably stems from the personal experiences of many visiting surgeons through the years who have seen an abundance of osteomyelitis, late-presenting open fractures, and badly performed internal fixations done by undertrained local and visiting surgeons in LMIC. SIGN, however, trains surgeons in the correct setup, indications, and techniques, and all reported X-rays are reviewed and commented on by SIGN staff if they show results that are not satisfactory. There is no reason that infection rates should be much higher in a low resource setting when well-trained surgeons with modern equipment have access to the basic requirements for surgery, such as autoclaves, antiseptic wash, and the right prophylactic antibiotics, as they increasingly have in even the poorest countries. In a large randomized study of prophylactic antibiotic use in Uganda, the rate of infection after inguinal hernia repair dropped from 7.5 to 0 % with correct antibiotic usage [28]. In our study prophylactic antibiotics reduced the risk of infection by 29 % (OR 0.71, 95 % CI: 0.55–0.91). A prospective multi-center study comparing results of a standardized IM nailing technique between a South African trauma center and European centers showed lower complication rates in South Africa and a near-identical infection rate despite more serious injuries in the South African patients [19]. One explanation for this can be a lower mean age of the patients in South Africa. Trauma is a growing epidemic among the young people of LMIC [4, 5]. This can also be seen in the SOSD, where the mean age is only 34.7 years.

The second interesting finding in this study was that fractures defined by the surgeon as a non-union prior to surgery had a 2.3 times increased adjusted risk of infection (OR 2.31, 95 % CI: 1.83–2.91). It is no news to orthopedic surgeons that operating to repair a non-union of the femur is a lot more work than operating on an acute fracture. The exposure is larger, the operating time longer, and the expected bleeding greater than with primary fracture surgery. In addition, it is possible that unknown factors regarding the biology of the fracture may be less favorable in non-unions. Though the definition of a non-union was at the discretion of the surgeons reporting the surgery, and might not reflect exactly the common definition of a fracture not healed at 6 months, it nevertheless is an expression of the surgeon’s doing surgery for a fracture at a delayed point in time when healing is not expected to occur. As such, in the authors’ opinion, this is an important finding, suggesting that outcomes are better when primary fracture surgery is done in LMIC, and in consequence that primary IM nailing should be offered for femur fractures in centers where the infrastructure and training of the surgeons allows. In uncomplicated closed tibia and humerus fractures, where there are good results of primary functional bracing that does not necessitate long-term hospital stay, this should probably still be the first-line treatment of choice [29–32].

Infection risk decreased with increasing country income level in this study, with higher middle-income countries having half the adjusted risk of infection that was seen in low-income countries (OR 0.49, 95 % CI: 0.30–0.81). Although this is a common preconception among surgeons, to our knowledge this has not been shown in an isolated study before. This would support the notion that the lack of infrastructure and the high prevalence of malnutrition and immunosuppression in low-income countries leads to an increased risk of infection in orthopedic trauma surgery. However, the numbers registered in higher middle-income countries are low, and these figures should be interpreted with caution. Even if these risk estimates are accurate, the risk of infection in low-income countries is still low and should not prohibit the use of modern orthopedic trauma surgery in these countries.

Tibia fractures had a twofold increased crude risk of infection and a 71 % increased adjusted risk of infection when an IM nail was used compared to antegrade nailing of the femur. As the adjusted risk of postoperative infection was adjusted for the increased incidence of open fractures of the tibia compared to the femur, this increase in infection risk is probably attributable to the subcutaneous localization of the tibia in contrast to the femur, which is surrounded by large, well-perfused muscles. No significant difference in the risk of infection was found between retrograde and antegrade nailing of the femur or between humerus and femur fractures.

There were 17 % open fractures in this study. This relatively high proportion of open fractures can possibly be seen as an expression of the severity of trauma in patients selected for IM nailing in LMIC. Open fractures had a 3.2 times increased risk of infection overall compared to closed fractures. The adjusted risk of infection rose with increasing severity of the injury from an OR of 1.9 for Gustilo grade 1 injuries to 7.6 for grade 3C injuries (Table 2). This is in line with other published reports and further supports that the data in the SOSD can be trusted [22, 33, 34]. No effect was seen on the adjusted risk of infection from age, gender, open reduction, number of distal locking screws, or method of reaming in this study.

There are some obvious limitations to this study, the most important being the low follow-up rate in the SOSD. Limited follow-up in studies in resource-constrained settings is a well-known problem. However, this might be necessary to accept if a large body of important information from poor countries is not to be kept out of the literature. In our above-mentioned earlier article validating the data in the SOSD in late 2010, we reported a follow-up rate of 18.1 % [21]. In that article we argued the case that the whole database can be used to estimate risk of infection based on the assumption that patients who have not returned for follow-up do not have infection. The present study builds on that assumption. We have also had to make several other assumptions that may not be correct. We have grouped superficial and deep infections together on the assumption that if they are reported they are serious enough to be of clinical importance, and we have assumed that if a patient returns with a complaint it will be registered in the SOSD. All this introduces uncertainty into the analyses and conclusions. However, in light of our former study, the large numbers in the SOSD, and the fact that we have analyzed the data both including and excluding patients without follow-up, we believe the reported figures give a good indication of where the true figures lie.

## Conclusions

This study seems to confirm the expected increase in postoperative infection risk in low-income countries compared to countries with higher income levels, and presumably better infrastructure, but the increase in infection rates was small (0.5–1.2 %). The overall infection rates were low, and well within acceptable levels, suggesting that it is safe to do IM nailing in low-income countries. The fact that operations for non-union have twice the risk of infection compared to primary fracture surgery further supports the use of IM nailing as the primary treatment for femur fractures in LMIC.
